# Distinct Patterns of Cerebral Extravasation by Evans Blue and Sodium Fluorescein in Rats

**DOI:** 10.1371/journal.pone.0068595

**Published:** 2013-07-05

**Authors:** Lola Fenghuei Yen, Vivi Chiali Wei, Eva Yuhua Kuo, Ted Weita Lai

**Affiliations:** 1 Graduate Institute of Clinical Medical Science, China Medical University, Taichung, Taiwan; 2 Translational Medicine Research Center, China Medical University Hospital, Taichung, Taiwan; University of Muenster, Germany

## Abstract

The Evans blue dye (EBD; 961 Da) and the sodium fluorescein dye (NaF; 376 Da) are commonly used inert tracers in blood-brain barrier (BBB) research. They are both highly charged low molecular weight (LMW) tracers with similar lipophobic profiles. Nevertheless, the EBD binds to serum albumin (69,000 Da) to become a high molecular weight (HMW) protein tracer when injected into the circulation, whereas the NaF remains an unbound small molecule in the circulation. In this study, rats were injected with equal doses of either EBD or NaF to monitor their blood and tissue distribution. The EBD was largely confined to the circulation with little accumulation in the peripheral organ and even less accumulation in the central tissue, whereas the NaF distributed more evenly between the blood and the peripheral organ but was also largely excluded from the central tissue. Importantly, the EBD crossed the BBB most effectively at the prefrontal cortex and the cerebellum, and most poorly at the striatum. In marked contrast, the NaF was evenly distributed throughout the brain. Finally, the EBD exhibited this same peculiar tissue distribution profile when administered by either bolus injection or slow infusion. Our study suggests that different regions of the brain are equally permeable to LMW inert dyes like the NaF, but are markedly different in permeability to HMW proteins such as EBD-labelled serum albumin.

## Introduction

The blood-brain barrier (BBB) restricts and thereby controls the exchanges of proteins and inert chemicals (those not permeable across endothelial cell membranes) between the brain and the cerebral circulation [1–4], and treatments that open the BBB are developed to facilitate brain delivery of high molecular weight (HMW) biologics (protein-based drugs) and low molecular weight (LMW) drug chemicals (small molecules) [5–9]. The simplest method for studying BBB permeability to HMW and LMW molecules is to measure tracer penetration into the brain when injected peripherally [10]. Of the tracers, the Evans blue dye (EBD; 961 Da) and the sodium fluorescein dye (NaF; 376 Da) are perhaps the most widely used in experimental animals. The EBD binds strongly to serum albumin (69,000 Da) to become a HMW protein tracer once in the circulation, whereas the NaF remains largely in the free, unbound and LMW form [10–13]. These properties make these two dyes ideal for studying BBB accessibility by HMW proteins and LMW chemicals, respectively.

The EBD becomes fully albumin-bound within 5 min post-i.v. bolus injection, and after then, no traces of the unbound EBD can be found in the blood [13]. It remains unclear exactly how much time, within the initial 5 min time frame, is required for EBD to bind to serum albumin. Hence, results from experiments in which the EBD was administered by bolus injection are called into question owing to the uncertainty over the degree to which bolus EBD entered the brain as an un-albumin-bound LMW dye, rather than as a presumed albumin-bound HMW tracer, before time is allowed for EBD to bind to albumin [14]. In this study, we reasoned that if HMW proteins and LMW chemicals take distinct routes across the BBB and into the brain, the difference could be detected by comparing their patterns of tissue distribution. Therefore, if bolus EBD entered the brain in part as a LMW chemical unbound to serum albumin, it would more closely resemble free LMW chemicals and take the LMW-way into the brain. In comparison, very slowly infused EBD would more closely resemble HMW proteins that take the HMW-way into the brain.

Despite the marked differences in functional molecular weight, results from experiments using either EBD or NaF are often mix-matched in the literature, with original and review articles going into details on the physiology, pathology, and pharmacology of the BBB but not clearly differentiating between BBB against HMW tracers and BBB against LMW tracers. In this study, we report the blood and tissue distribution of the EBD and the NaF when injected into experimental rats. In particular, we used statistical pattern analysis to differentiate between the routes taken by either dyes into the brain.

## Materials and Methods

### Animals

Sixty-six male Sprague-Dawley rats (P49-P71; 260-400g) were used in this study. They were housed and cared for in accordance with the Institutional Guidelines of the China Medical University for the Care and Use of Experimental Animals (IGCMU-CUEA), and all experimental procedures were approved by the Institutional Animal Care and Use Committee (IACUC) of the China Medical University (Taichung, Taiwan) (Protocol No. 101-274-N).

### Dye injection and sample collection

Each rat was anesthetized with urethane, and its femoral vein and artery were cannulated to facilitate dye injection and blood collection. Body temperature was monitored at all times, and controlled at ~37 ^°^C by an automated heating pad that received active feedback from the rectal probe. The rats received either (1) EBD by i.v. bolus injection, (2) NaF by i.v. bolus injection, or (3) EBD by i.v. slow infusion at a rate of 1 ml/hr. The dyes were prepared as 4% solutions (except when indicated otherwise) dissolved in 0.9% saline, and injected at a dose of 2 ml/kg into the cannulated femoral vein. Blood samples were collected from the femoral artery prior to and at 5, 30, 60, 90, and 120 min post-dye injection. Following the final blood collection, each rat was euthanized under urethane-anesthesia by perfusion with 0.9% saline to rid the circulation of dye (as described previously [15]). Thereafter, the tissue parenchyma containing only the extravasated dye was collected.

### Brain regions

**Figure 1:**
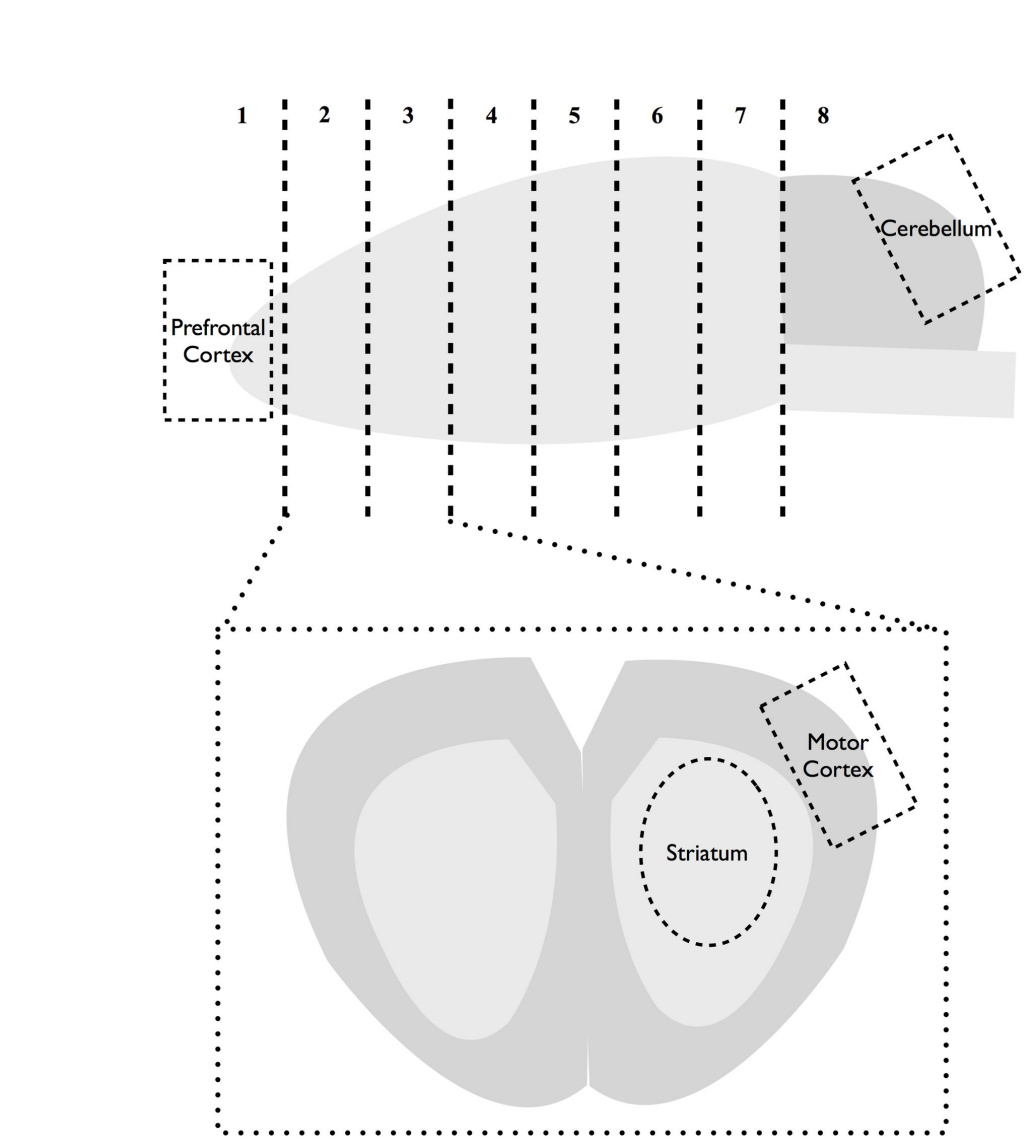
Illustration of the brain regions collected for analysis.

The isolated brain was coronal sectioned into 2-mm slices, with cutting points corresponding to (distance from bregma): section 1: +5.6mm, section 2: +3.6mm, section 3: +1.6mm, section 4: -0.4mm, section 5: -2.4mm, and section 6: -4.4mm. As illustrated in Figure **1**, the prefrontal cortex was isolated from section 1, and the motor cortex and the striatum were isolated from sections 2 and 3. The cerebellum was conservatively isolated to avoid collection of the brain stem underneath.

### Sample processing and photospectrometry

Blood and tissue samples were processed using the dye-extraction method described previously, which completely recovered dyes from binding to tissue, serum albumin, and other proteins [16]. The blood samples were centrifuged at 10,000 rpm for 10 min, and the supernatants were mixed with 1: 3 volume of 50% trichloroacetic acid (TCA; dissolved in 0.9% saline) and centrifuged again (10,000 rpm for 10 min). The final supernatants were diluted with 1: 300 volume of 50% TCA, and then 1: 3 volume of 95% ethanol prior to photospectrometric determination of EBD (620nm excitation/680nm emission) and NaF (440nm excitation/525nm emission) fluorescence. The brain and liver tissues were dry-weighted, homogenized in 1: 3 volume of 50% TCA, centrifuged (10,000 rpm for 10 min), and the supernatants were diluted with 1: 3 volume of 95% ethanol prior to photospectrometric determination of EBD and NaF fluorescence.

### Data presentation and statistical analysis

Data were presented as mean±SEM. The change in blood concentration of dye in matched subjects was analyzed by TWO-WAY repeated measures ANOVA, followed by Sidak’s multiple comparisons test. Paired comparisons between blood and liver dye contents of matched subjects were done with paired t test. Comparisons of tissue dye contents between animals receiving either EBD or NaF were done with unpaired t test. Pattern analyses that examines regional specificity of dye distribution in matched brain tissues were done by ONE-WAY repeated measures ANOVA, followed by multiple comparisons tests with Tukey’s or Holm-Sidak. Cross-comparisons of dye distribution patterns between two groups of animals with matched brain tissues were done using TWO-WAY repeated measures ANOVA.

## Results

**Figure 2:**
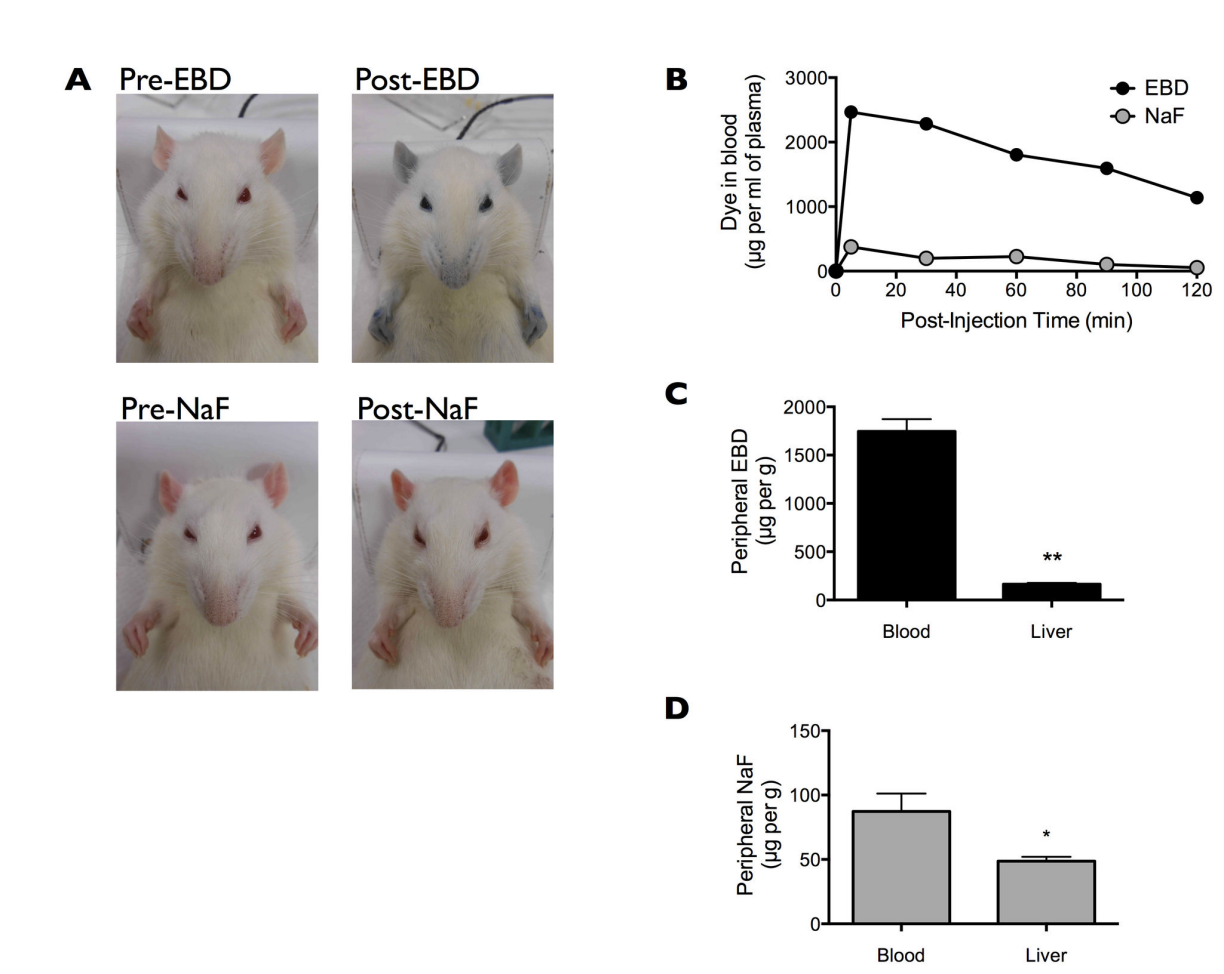
Peripheral accumulation of Evans blue dye (EBD) and sodium fluorescein dye (NaF). (A) Representative images of rats before and after receiving EBD or NaF at equal doses (80 mg/kg i.v. bolus into the femoral vein). Post-injection images were taken at 2 h post-injection. (B) Blood concentration of EBD or NaF prior to and at 5, 30, 60, 90, and 120 min post-injection. Samples were collected from a cannulated femoral artery. (C) Peripheral EBD concentration in the blood and the liver at 120 min post-injection. Data are shown as mean±SEM. **p<0.01, comparison by paired t test. (D) Peripheral NaF concentration in the blood and the liver at 120 min post-injection. Data are shown as mean±SEM. *p<0.05, comparison by paired t test.

Consistent with previous reports, EBD (80 mg/kg i.v. bolus) rendered the rat eyes, ears, nose, and paws dark blue, and this blue coloration persisted for the 2 h duration of the experiment (Figure **2A**, top panels). In comparison, NaF (80 mg/kg i.v. bolus) turned the rat orange, but the coloration was much less apparent and appeared to quickly subside over the 2 h course of the experiment (Figure **2A**, bottom panels).

Given that EBD but not NaF binds strongly to serum albumin [10–13], the peripheral distribution of the two dyes should be quite distinct. EBD injection resulted in a substantial accumulation of the dye in the blood, especially in comparison to the injection of the same-dose NaF (Figure 2B). Moreover, direct comparison between blood and liver dye contents showed that EBD resided largely in the blood (1746 ± 127 µg/ml) rather than in the peripheral organ (164 ± 13 µg/g) (Figure 2C). In contrast, NaF was more evenly distributed between the blood (87 ± 14 µg/ml) and the peripheral organ (49 ± 4 µg/g) (Figure 2D). Our findings are in line with the ample evidence showing that EBD but not NaF binds strongly to serum albumin, and thus EBD but not NaF resided predominantly in the blood.

**Figure 3:**
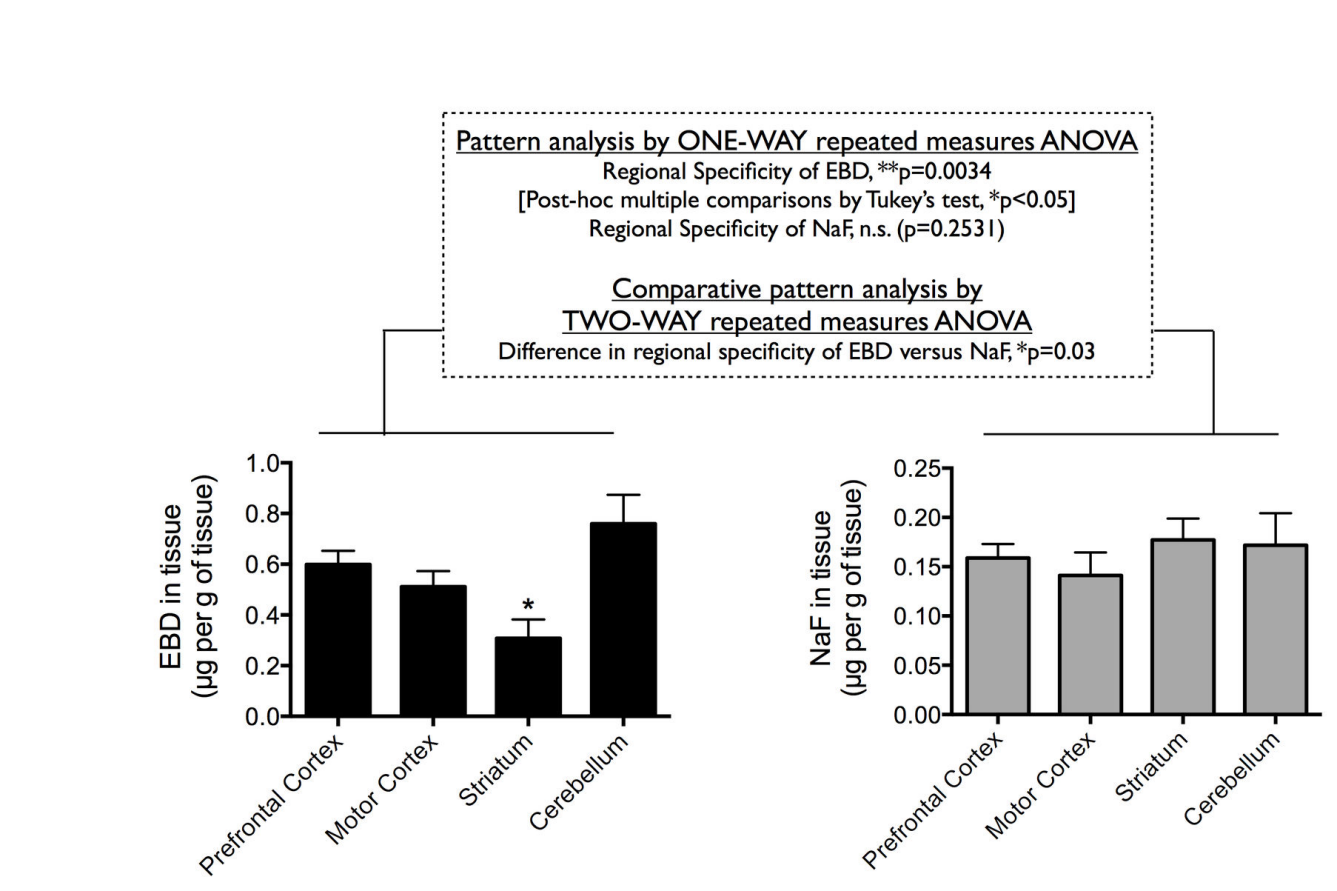
Central extravasation of Evans blue dye (EBD) and sodium fluorescein dye (NaF). Regional brain distribution of extravasated EBD (left panel) or NaF (right panel) in the prefrontal cortex, the motor cortex, the striatum, and the cerebellum following equal dose injections (80 mg/kg i.v. bolus into the femoral vein). Data are shown as mean±SEM. Statistics used are described in the figure.

The BBB is known to restrict extravasation of inert tracers such as EBD and NaF into the brain [10], and in our experiment, these two dyes were indeed largely excluded from the brain parenchyma (Figure 3), with concentrations ranging from 0.2–0.9 µg per g of brain tissue compared to >40 µg per g of liver tissue. Moreover, and consistent with higher circulating EBD concentration and lower circulating NaF concentration (Figure 2B), EBD extravasated at a much higher concentration into the brain than that by NaF in most brain regions studied (Figure 3). These data support the notion that the BBB restricts these inert tracers into the brain, and that the concentration of dye that is extravasated across the BBB may be dependent on the concentration of the dye in the circulation.

The primary objective of this study was to examine whether the two most commonly used tracers for BBB research, EBD and NaF, exhibits distinct patterns of regional brain distribution when injected into the circulation. This was especially that EBD binds to serum albumin (69,000 Da) to effectively become a HMW protein tracer, whereas NaF remains mostly in the LMW chemical form (376 Da). Interestingly and surprisingly, pattern analysis by ONE-WAY repeated measures ANOVA found significant regional specificity for EBD extravasation (**p=0.0034; followed by post-hoc multiple comparisons by Tukey’s test, *p<0.05) (Figure **3**, left panel). In particular, EBD appeared to extravasate most effectively at the prefrontal cortex and the cerebellum (albeit not significant), and least effectively at the striatum (*p<0.05, compared to extravasation at other tissues). In marked comparison, NaF was evenly distributed between different brain regions, including the prefrontal cortex, the motor cortex, the striatum, and the cerebellum (not significant, when compared by ONE-WAY repeated measures ANOVA) (Figure **3**, right panel). Indeed, comparative pattern analysis that compared regional brain distribution of the two dyes by TWO-WAY repeated measures ANOVA found significant difference between the patterns of EBD and NaF brain distribution (*p=0.0311) (Figure 3).

**Figure 4:**
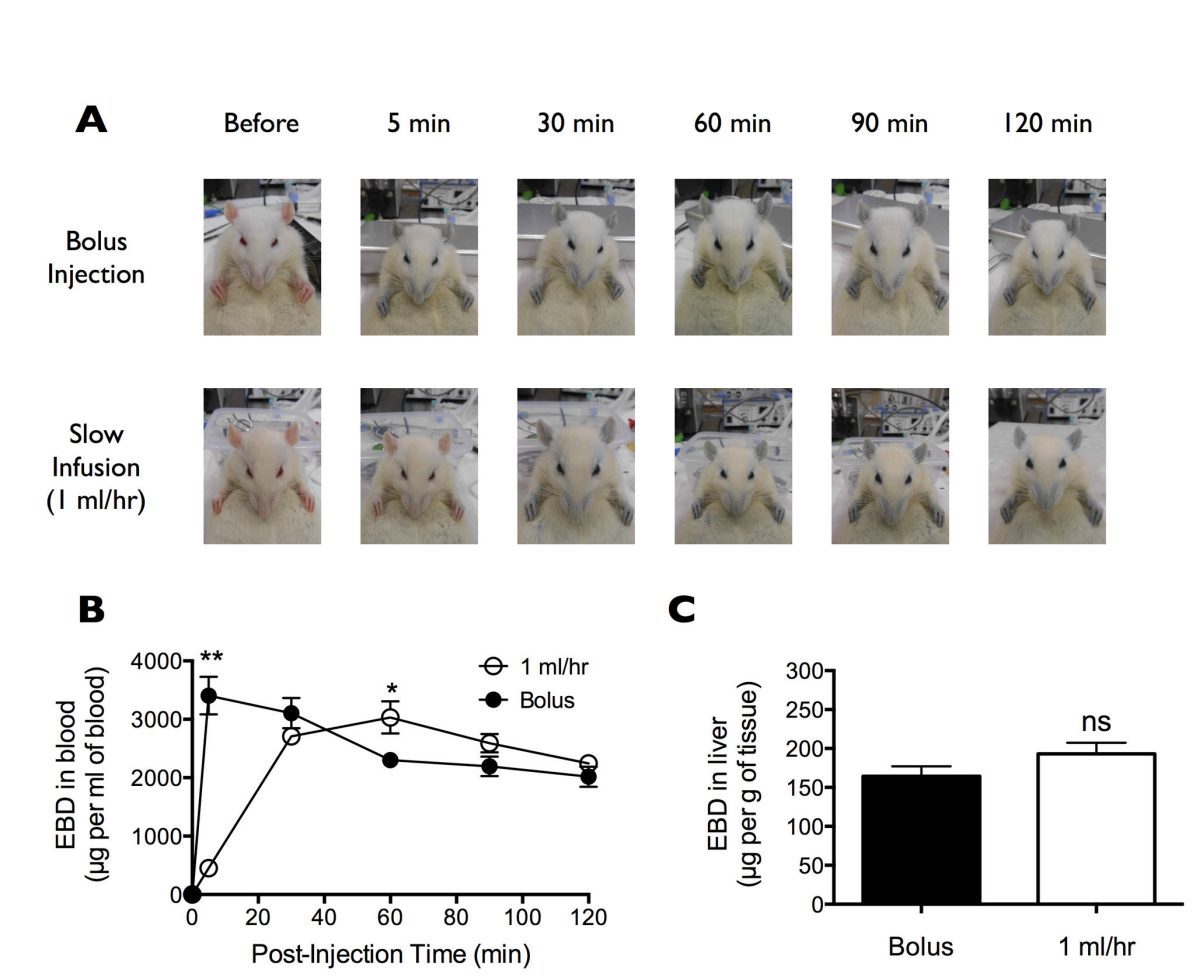
Peripheral accumulation of Evans blue dye (EBD) following bolus injection and slow infusion. (A) Representative images of rats before and after receiving 4% EBD via bolus injection or slow infusion (1 ml/hr) at equal doses (2 ml/kg i.v. into the femoral vein). (B) Blood concentration of EBD prior to and at 5, 30, 60, 90, and 120 min post-bolus injection or slow infusion. Samples were collected from a cannulated femoral artery. Data are shown as mean±SEM. *p<0.05 and **p<0.01, comparison by TWO-WAY repeated measures ANOVA followed by Sidak’s multiple comparisons test. (C) Peripheral EBD concentration in the liver at 120 min post-bolus injection or slow infusion. Data are shown as mean±SEM. n.s., no significant difference when compared by unpaired t test.

Although the EBD is often administered to experimental animals by i.v. bolus injection, the interpretation of results from these studies are called into questions over concerns that EBD may enter the brain in the free-unbound LMW form before time is allowed for binding to the HMW serum albumin [14]. Because a 5 min time period is sufficient for all EBD molecules to fully bind to serum albumin [13], we examined the peripheral/central distribution of EBD when infused at a rate (1 ml/hr i.v.) that takes more than 5 min to plateau. While bolus EBD fully colored the rat blue within 5 min post-injection, slowly infused EBD took ~30 min before saturation of its blue appearance (Figure 4A). Moreover, bolus EBD spiked in the blood immediately following injection and then decreased over the 2 h course of the experiment, whereas slowly infused EBD took about 30 min before reaching a plateau in blood concentration (Figure 4B). These data suggest that slow infusion of EBD at a rate of 1 ml/hr should satisfy the criteria for full binding of EBD to serum albumin prior to leakage into peripheral organs and across the BBB.

**Figure 5:**
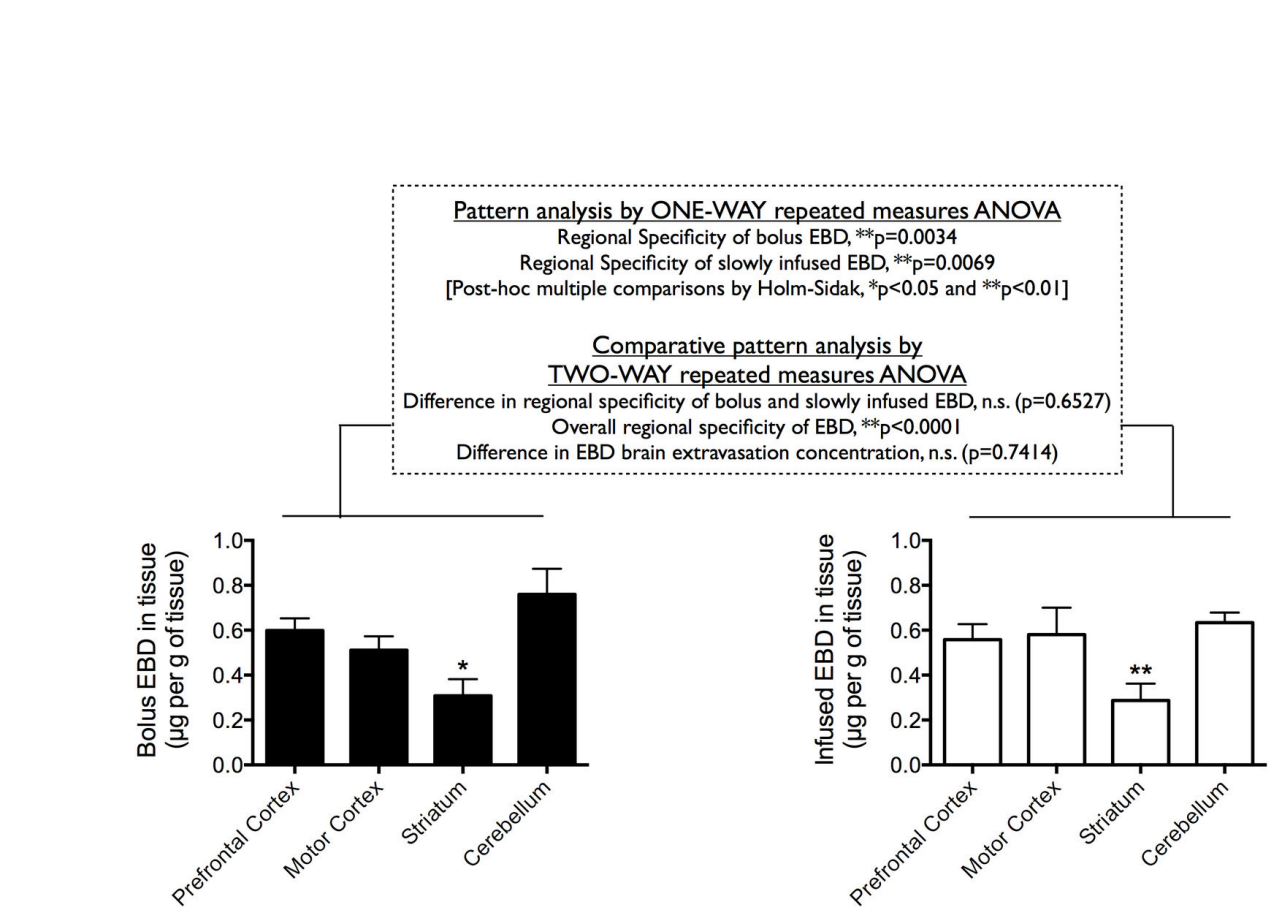
Central extravasation of Evans blue dye (EBD) following bolus injection and slow infusion. Regional brain distribution of extravasated EBD in the prefrontal cortex, the motor cortex, the striatum, and the cerebellum following bolus injection (left panel) or slow infusion (right panel) (80 mg/kg i.v. into the femoral vein). Data are shown as mean±SEM. Statistics used are described in the figure.

Taking into account the marked difference in EBD and NaF extravasation into peripheral and central tissues, we reasoned that if bolus EBD indeed extravasated in part as a pure LMW dye (yet unbound to serum albumin), it would exhibit a slightly different tissue extravasation concentration/pattern profile compared to that of slowly infused EBD. Importantly, EBD administered by either bolus injection or slow infusion exhibited similar level of accumulation into the liver (Figure 4C) and different regions of the brain (Figure 5). Here, pattern analysis by ONE-WAY repeated measures ANOVA found significant regional specificity of EBD distribution in the rat brain following either bolus injection (**p=0.0034) or slow infusion (**p=0.0069) (Figure 5). In both cases, post-hoc multiple comparisons by Holm-Sidak test found significantly higher extravasation of EBD into the prefrontal cortex, the motor cortex, and the cerebellum, compared to EBD into the striatum. Importantly, albeit an overall significant regional specificity (**p<0.0001), comparative pattern analysis by TWO-WAY repeated measures ANOVA found no significant difference when comparing the patterns of EBD brain distribution following bolus injection and slow infusion (n.s., p=0.6527). Our findings suggest that even when injected in a single bolus, EBD took the “HMW-way” into the brain, rather than the “LMW-way” taken by NaF.

**Figure 6:**
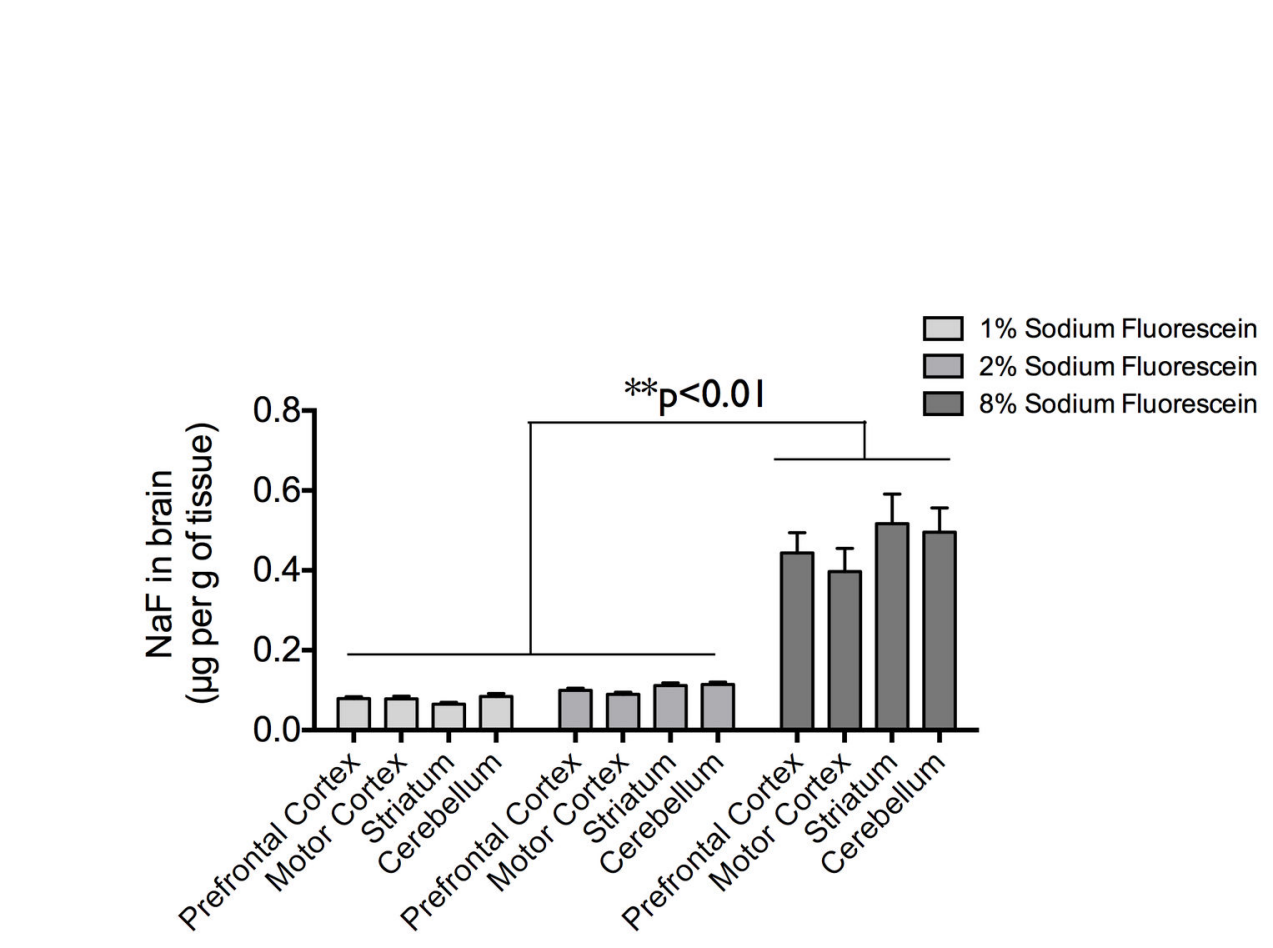
Central extravasation of sodium fluorescein dye (NaF) in a dose-dependent manner. Regional brain distribution of extravasated NaF in the prefrontal cortex, the motor cortex, the striatum, and the cerebellum following bolus injection (2 ml/kg i.v. into the femoral vein) of 1%, 2%, or 8% NaF. Data are shown as mean±SEM. **p<0.01 when compared by TWO-WAY repeated measures ANOVA followed by Tukey’s multiple comparisons test.

The distinct pattern of cerebral extravasation by EBD, with minimal extravasation into the striatal parenchyma compared to other brain regions, was observed when the dye was administered either by bolus injection or slow infusion. This was in marked contrast with NaF that extravasated somewhat evenly throughout different brain regions. However, this contrasting difference in the dye extravasation pattern could also be due to the difference in the blood concentration and hence difference in the amount of dye available for extravasation. To test this possibility, we administered different doses of NaF (1%, 2%, and 8% at 2 ml/kg i.v.) by bolus injection (Figure 6). We found that the amount of NaF extravasated into the brain is dose-dependent on the amount of NaF administered into the bloodstream (TWO-WAY repeated measures ANOVA followed by post-hoc multiple comparisons using Tukey’s test, **p<0.01). In fact, the amount of 8% NaF extravasated is comparable to the amount of 4% EBD extravasated. Nevertheless, the same pattern of cerebral extravasation, with no shortage of striatal extravasation, was observed regardless of the NaF dose injected. This suggest that the peculiar pattern of cerebral extravasation by 4% EBD but not 4% NaF was not due to the higher extravasation of the former.

## Discussion

The BBB safeguards the brain against unwanted exchanges of HMW proteins and LMW inert substances, and research in BBB has important physiological, pathological, and pharmacological implications. Physiological changes of the BBB occur throughout development [15] and during emotional responses [17–19] and other behaviors [19,20], and pathological changes of the BBB result from ischemic/hemorrhagic stroke [21,22], seizure [23], Alzheimer’s disease [24–26], Parkinson’s disease [27], HIV-associated neurocognitive disorders [28,29], multiple sclerosis [30], and obesity [20]. In addition, because the BBB is a major obstacle for drug delivery into the brain, its pharmacology has been investigated widely [8,9,31–35]. Nevertheless, the literature has in large failed to emphasize the differences between the HMW-way and the LMW-way across the BBB into the brain.

We report here that the HMW-way and the LMW-way across the BBB represent two distinct routes into the brain, and such distinction can be made by comparing the regions of the brain most accessible to the respective substance. For instance, HMW proteins marked by EBD entered the brain most efficiently from the prefrontal cortex and the cerebellum, and least effectively from the striatum. In comparison, LMW inert tracers like NaF was evenly distributed across different brain regions. The reason for the difference warrants future research. One likely explanation is that different regions of the BBB have different systems for actively importing and/or exporting HMW proteins, whereas LMW substances are allowed to passively move across in a restricted manner. Another possibility is that the BBB has brain region-specific different-sized physical barrier that bars HMW proteins from moving into and out of the brain, and that these brain-region-specific large barriers are circumvented by LMW inert tracers. Whatever the reason behind the regional-dependency of the HMW-way by not the LMW-way, our data re-emphasize the notion that the BBB is substance/molecular weight-specific, and should not be generalized otherwise.

Based on these distinct patterns of brain distribution, we reasoned that bolus and slowly infused EBD both entered the brain via the HMW-way, and this is in line with the notion that EBD binds strongly to serum albumin immediately following bolus injection [10–13]. This work advocates the use of bolus EBD in BBB research, and ameliorates the concerns that EBD administered by i.v. bolus, the most widely used route of administration in EBD-based BBB studies, may behave like a LMW dye rather than a HMW protein tracer [14]. Moreover, this provides a good example of how pattern analysis of EBD brain distribution can be used to determine whether a chemical substance enters the brain via the HMW/EBD-way or the LMW/NaF-way.

Disruption (or closure) of the BBB, as part of a physiological response, resulting from a disease, or induced by a pharmacological agent, could result in increased or decreased access to either or both of the HMW (EBD-labelled) protein and the LMW inert substances (such as NaF). Thus, pharmacological agents designed to selectively facilitate or prevent the HMW/EBD-way or the LMW/NaF-way across the BBB may have advantages over agents that are less selective. In any case, these two different routes of entrance into the brain emphasize the need to categorize the BBB depending on the substance in question.

## References

[1] Reese TS, Karnovsky MJ (1967) Fine structural localization of a blood-brain barrier to exogenous peroxidase. J Cell Biol 34: 207-217. doi:10.1083/jcb.34.1.207. PubMed: 6033532. 6033532 10.1083/jcb.34.1.207 PMC2107213

[2] Brightman MW, Reese TS (1969) Junctions between intimately apposed cell membranes in the vertebrate brain. J Cell Biol 40: 648-677. doi:10.1083/jcb.40.3.648. PubMed: 5765759. 5765759 10.1083/jcb.40.3.648 PMC2107650

[3] Marchi N, Cavaglia M, Fazio V, Bhudia S, Hallene K et al. (2004) Peripheral markers of blood-brain barrier damage. Clin Chim Acta 342: 1-12. doi:10.1016/j.cccn.2003.12.008. PubMed: 15026262. 15026262 10.1016/j.cccn.2003.12.008

[4] Weiss N, Miller F, Cazaubon S, Couraud PO (2009) The blood-brain barrier in brain homeostasis and neurological diseases. Biochim Biophys Acta 1788: 842-857. doi:10.1016/j.bbamem.2008.10.022. PubMed: 19061857. 19061857 10.1016/j.bbamem.2008.10.022

[5] Doolittle ND, Miner ME, Hall WA, Siegal T, Jerome E et al. (2000) Safety and efficacy of a multicenter study using intraarterial chemotherapy in conjunction with osmotic opening of the blood-brain barrier for the treatment of patients with malignant brain tumors. Cancer 88: 637-647. doi:10.1002/(SICI)1097-0142(20000201)88:3. PubMed: 10649259. 10649259 10.1002/(sici)1097-0142(20000201)88:3<637::aid-cncr22>3.0.co;2-y

[6] Rapoport SI (2001) Advances in osmotic opening of the blood-brain barrier to enhance CNS chemotherapy. Expert Opin Investig Drugs 10: 1809-1818. doi:10.1517/13543784.10.10.1809. PubMed: 11772287. 10.1517/13543784.10.10.1809 11772287

[7] Angelov L, Doolittle ND, Kraemer DF, Siegal T, Barnett GH et al. (2009) Blood-brain barrier disruption and intra-arterial methotrexate-based therapy for newly diagnosed primary CNS lymphoma: a multi-institutional experience. J Clin Oncol 27: 3503-3509. doi:10.1200/JCO.2008.19.3789. PubMed: 19451444. 19451444 10.1200/JCO.2008.19.3789 PMC2717756

[8] Carman AJ, Mills JH, Krenz A, Kim DG, Bynoe MS (2011) Adenosine receptor signaling modulates permeability of the blood-brain barrier. J Neurosci 31: 13272-13280. doi:10.1523/JNEUROSCI.3337-11.2011. PubMed: 21917810. 21917810 10.1523/JNEUROSCI.3337-11.2011 PMC3328085

[9] Chen KB, Wei VC, Yen LF, Poon KS, Liu YC et al. (2013) Intravenous mannitol does not increase blood-brain barrier permeability to inert dyes in the adult rat forebrain. Neuroreport 24: 303-307. doi:10.1097/WNR.0b013e32835f8acb. PubMed: 23426109. 23426109 10.1097/WNR.0b013e32835f8acb

[10] Kaya M, Ahishali B (2011) Assessment of permeability in barrier type of endothelium in brain using tracers: Evans blue, sodium fluorescein, and horseradish peroxidase. Methods Mol Biol 763: 369-382. doi:10.1007/978-1-61779-191-8_25. PubMed: 21874465. 21874465 10.1007/978-1-61779-191-8_25

[11] Saria A, Lundberg JM (1983) Evans blue fluorescence: quantitative and morphological evaluation of vascular permeability in animal tissues. J Neurosci Methods 8: 41-49. doi:10.1016/0165-0270(83)90050-X. PubMed: 6876872. 6876872 10.1016/0165-0270(83)90050-x

[12] Patterson CE, Rhoades RA, Garcia JG (1992) Evans blue dye as a marker of albumin clearance in cultured endothelial monolayer and isolated lung. J Appl Physiol 72: 865-873. doi:10.1063/1.351760. PubMed: 1568982. 1568982 10.1152/jappl.1992.72.3.865

[13] Wolman M, Klatzo I, Chui E, Wilmes F, Nishimoto K et al. (1981) Evaluation of the dye-protein tracers in pathophysiology of the blood-brain barrier. Acta Neuropathol 54: 55-61. doi:10.1007/BF00691332. PubMed: 7234328. 7234328 10.1007/BF00691332

[14] Kozler P, Pokorný J (2003) Altered blood-brain barrier permeability and its effect on the distribution of Evans blue and sodium fluorescein in the rat brain applied by intracarotid injection. Physiol Res 52: 607-614. PubMed: 14535837. 14535837

[15] Chen KB, Kuo EY, Poon KS, Cheng KS, Chang CS et al. (2012) Increase in Evans blue dye extravasation into the brain in the late developmental stage. Neuroreport 23: 699-701. doi:10.1097/WNR.0b013e3283556dcc. PubMed: 22729097. 22729097 10.1097/WNR.0b013e3283556dcc

[16] Uyama O, Okamura N, Yanase M, Narita M, Kawabata K et al. (1988) Quantitative evaluation of vascular permeability in the gerbil brain after transient ischemia using Evans blue fluorescence. J Cereb Blood Flow Metab 8: 282-284. doi:10.1038/jcbfm.1988.59. PubMed: 3343300. 3343300 10.1038/jcbfm.1988.59

[17] Skultétyová I, Tokarev D, Jezová D (1998) Stress-induced increase in blood-brain barrier permeability in control and monosodium glutamate-treated rats. Brain Res Bull 45: 175-178. doi:10.1016/S0361-9230(97)00335-3. PubMed: 9443836. 9443836 10.1016/s0361-9230(97)00335-3

[18] Esposito P, Gheorghe D, Kandere K, Pang X, Connolly R et al. (2001) Acute stress increases permeability of the blood-brain-barrier through activation of brain mast cells. Brain Res 888: 117-127. doi:10.1016/S0006-8993(00)03026-2. PubMed: 11146058. 11146058 10.1016/s0006-8993(00)03026-2

[19] Belova I, Jonsson G (1982) Blood-brain barrier permeability and immobilization stress. Acta Physiol Scand 116: 21-29. doi:10.1111/j.1748-1716.1982.tb10594.x. PubMed: 6891559. 6891559 10.1111/j.1748-1716.1982.tb10594.x

[20] Banks WA (2012) Role of the blood-brain barrier in the evolution of feeding and cognition. Ann N Y Acad Sci 1264: 13-19. doi:10.1111/j.1749-6632.2012.06568.x. PubMed: 22612379. 22612379 10.1111/j.1749-6632.2012.06568.x PMC3464352

[21] ElAli A, Doeppner TR, Zechariah A, Hermann DM (2011) Increased blood-brain barrier permeability and brain edema after focal cerebral ischemia induced by hyperlipidemia: role of lipid peroxidation and calpain-1/2, matrix metalloproteinase-2/9, and RhoA overactivation. Stroke 42: 3238-3244. doi:10.1161/STROKEAHA.111.615559. PubMed: 21836084. 21836084 10.1161/STROKEAHA.111.615559

[22] Manaenko A, Chen H, Kammer J, Zhang JH, Tang J (2011) Comparison Evans Blue injection routes: Intravenous versus intraperitoneal, for measurement of blood-brain barrier in a mice hemorrhage model. J Neurosci Methods 195: 206-210. doi:10.1016/j.jneumeth.2010.12.013. PubMed: 21168441. 21168441 10.1016/j.jneumeth.2010.12.013 PMC3026886

[23] Librizzi L, Noè F, Vezzani A, de Curtis M, Ravizza T (2012) Seizure-induced brain-borne inflammation sustains seizure recurrence and blood-brain barrier damage. Ann Neurol 72: 82-90. doi:10.1002/ana.23567. PubMed: 22829270. 22829270 10.1002/ana.23567

[24] Bell RD, Winkler EA, Singh I, Sagare AP, Deane R et al. (2012) Apolipoprotein E controls cerebrovascular integrity via cyclophilin A. Nature 485: 512-516. PubMed: 22622580. 22622580 10.1038/nature11087 PMC4047116

[25] Castellano JM, Deane R, Gottesdiener AJ, Verghese PB, Stewart FR et al. (2012) Low-density lipoprotein receptor overexpression enhances the rate of brain-to-blood Abeta clearance in a mouse model of beta-amyloidosis. Proc Natl Acad Sci U S A. 10.1073/pnas.1206446109 PMC3458349 22927427

[26] Zlokovic BV (2011) Neurovascular pathways to neurodegeneration in Alzheimer’s disease and other disorders. Nat Rev Neurosci 12: 723-738. PubMed: 22048062. 22048062 10.1038/nrn3114 PMC4036520

[27] Pisani V, Stefani A, Pierantozzi M, Natoli S, Stanzione P et al. (2012) Increased blood-cerebrospinal fluid transfer of albumin in advanced Parkinson’s disease. J Neuroinflammation 9: 188. doi:10.1186/1742-2094-9-188. PubMed: 22870899. 22870899 10.1186/1742-2094-9-188 PMC3441323

[28] Gorantla S, Poluektova L, Gendelman HE (2012) Rodent models for HIV-associated neurocognitive disorders. Trends Neurosci 35: 197-208. doi:10.1016/j.tins.2011.12.006. PubMed: 22305769. 22305769 10.1016/j.tins.2011.12.006 PMC3294256

[29] Zhong Y, Zhang B, Eum SY, Toborek M (2012) HIV-1 Tat triggers nuclear localization of ZO-1 via Rho signaling and cAMP response element-binding protein activation. J Neurosci 32: 143-150. doi:10.1523/JNEUROSCI.4266-11.2012. PubMed: 22219277. 22219277 10.1523/JNEUROSCI.4266-11.2012 PMC3566645

[30] Arima Y, Harada M, Kamimura D, Park JH, Kawano F et al. (2012) Regional neural activation defines a gateway for autoreactive T cells to cross the blood-brain barrier. Cell 148: 447-457. doi:10.1016/j.cell.2012.01.022. PubMed: 22304915. 22304915 10.1016/j.cell.2012.01.022

[31] Hjouj M, Last D, Guez D, Daniels D, Sharabi S et al. (2012) MRI study on reversible and irreversible electroporation induced blood brain barrier disruption. PLOS ONE 7: e42817. doi:10.1371/journal.pone.0042817. PubMed: 22900052. 22900052 10.1371/journal.pone.0042817 PMC3416789

[32] Joshi S, Ergin A, Wang M, Reif R, Zhang J et al. (2011) Inconsistent blood brain barrier disruption by intraarterial mannitol in rabbits: implications for chemotherapy. J Neuro Oncol 104: 11-19. doi:10.1007/s11060-010-0466-4. PubMed: 21153681. 10.1007/s11060-010-0466-4 PMC4013427 21153681

[33] Malpass K (2011) New methods to permeabilize the blood-brain barrier. Nat Rev Neurol 7: 597. doi:10.1038/nrneurol.2011.171. PubMed: 22009285. 22009285 10.1038/nrneurol.2011.161

[34] Tung YS, Marquet F, Teichert T, Ferrera V, Konofagou EE (2011) Feasibility of noninvasive cavitation-guided blood-brain barrier opening using focused ultrasound and microbubbles in nonhuman primates. Appl Phys Lett 98: 163704. doi:10.1063/1.3580763. PubMed: 21580802. 21580802 10.1063/1.3580763 PMC3094460

[35] Marquet F, Tung YS, Teichert T, Ferrera VP, Konofagou EE (2011) Noninvasive, transient and selective blood-brain barrier opening in non-human primates in vivo. PLOS ONE 6: e22598. doi:10.1371/journal.pone.0022598. PubMed: 21799913. 21799913 10.1371/journal.pone.0022598 PMC3142168

